# Morphometry of the basal cell layer of oral leukoplakia and oral squamous cell carcinoma using computer-aided image analysis

**DOI:** 10.4103/0973-029X.80034

**Published:** 2011

**Authors:** T Smitha, P Sharada, HC Girish

**Affiliations:** *Department of Oral and Maxillofacial Pathology, V. S. Dental College and Hospital, K. R. Road, VV Puram, Bangalore, India*; 1 *AECS Maruti College of Dental Sciences and Research Centre, Bannerghatta Road, Bangalore, India*; 2 *Rajarajeshwari Dental College, Kumbalgodu, Ramohalli Cross, Bangalore, India*

**Keywords:** Image analysis, leukoplakia, morphometry, parameters

## Abstract

**Objectives:**

To study and compare the changes in nuclear and cellular size, shape and nuclear–cytoplasmic ratio of the cells in the basal layer of oral leukoplakia and well-differentiated oral squamous cell carcinoma (SCC) with normal buccal mucosa, using computer-aided image analysis in tissue sections.

**Study design:**

This was a retrospective study conducted on tissue sections on a total number of 70 cases to determine the various morphometric parameters. The data collected in this study were analyzed statistically by computing descriptive statistics, viz., percentage, mean, standard deviation, standard error of mean, 95% confidence interval for mean. The difference in the control and study groups for various diagnostic variables was compared by means of analysis of variance (ANOVA), Student’s *t*-test for independent samples, wherever applicable. Mann–Whitney U-test and Kruskal–Wallis test were used where the data were found to be asymmetrical and the standard deviations were also different. The results were considered statistically significant whenever *P* ≤ 0.05.

**Results:**

Our results were significant for the morphometric parameter, size. The values of nuclear perimeter and area, cellular perimeter and area increased gradually from the normal buccal mucosa to leukoplakia, reaching the highest value in SCC. There was statistically significant difference in the nuclear and cellular areas to differentiate between leukoplakia and squamous cell carcinoma. Two variables which were used to study the shape, “form perimeter (PE)” and “contour index (CI)”, showed significant difference between normal buccal mucosa and leukoplakia and between normal buccal mucosa and SCC. The morphometric parameter, nuclear–cytoplasmic ratio, in our results showed an increase in leukoplakia and SCC compared to normal buccal mucosa, but the difference was not significant between leukoplakia and SCC.

**Conclusion:**

The morphometric parameter, size, was useful to differentiate between normal, potentially malignant leukoplakia and SCC.

## INTRODUCTION

Oral squamous cell carcinoma (SCC) is recognized as the most common malignant epithelial neoplasm of the oral cavity, resulting from genetic damage, leading to uncontrolled cell proliferation of damaged cells.[1] In the course of its progression, visible changes are taking place at the cellular level (*atypical*) and at the resultant tissue level (*epithelial dysplasia*).[2] The sum total of these physical and morphological alterations is of diagnostic and prognostic relevance and is designated as precancerous changes. Oral leukoplakia is the best known precursor lesion. Earlier, clinical investigations had shown the rate of malignant change in leukoplakia ranging between 3% and 6%.[3] The latter studies suggest a malignant transformation potential of 4%, and is higher in specific clinical subtypes or phases of leukoplakia.[4] In two studies from India carried out by Gupta *et al*. and Silvennan *et al*., annual malignant transformation rates of 0.3% and 0.06%, respectively, have been reported.[5] The dilemma in managing patients with potentially malignant oral lesions and field change is of deciding which mucosal lesions or areas will progress to carcinoma.[6] From the vast literature on molecular markers in oral precancer, no reliable prognostic markers for risk assessment in putatively premalignant lesions have emerged.[7] However, histopathologically assessed severity of oral epithelial dysplasia (OED) is considered as the “gold standard” for the prediction of malignant transformation of precancerous lesions.[8]

Dysplasia comprises a loss in the uniformity of the individual cells as well as a loss in their architectural orientation. Dysplastic cells exhibit considerable pleomorphism (variation in the size and shape) and often possess hyperchromatic nuclei which are abnormally large for the size of the cell. The nuclear–cytoplasmic ratio increases from 1:4 to 1:1 at the expense of the cytoplasmic volume.[2]

There is, therefore, a substantial need to improve the histologic assessment of epithelial dysplasia. Pathologists differ in the emphasis they place on particular histopathologic features, and the interpretation of dysplasia varies from one pathologist to another. The wide variation between the observers in the subjective evaluation has been commented on by several authors and more objective means of evaluation are constantly sought for.[9–13] The interest then turned toward applying sophisticated technique of computer-assisted morphometry to investigate the cellular and nuclear changes in correlation with the histological behavior of the lesions.[14] It is a method of assessing the computerized images of histological stained sections. Several variables observable in microscopic images are amenable to morphometric analysis. The results have been more reliable, objective and reproducible.

The present study was conducted to observe the morphological features like cell area (CA), nuclear area (NA), cell perimeter (CP), nuclear perimeter (NP), and to assess the shape and nuclear–cytoplasmic ratio (N/C) in the *basal cell layer in normal buccal mucosa, leukoplakia* and *SCC*, and to see if any consistent changes in these features existed between them. The study group comprised a total of 70 cases, of which 10 cases were of normal buccal mucosa (control), 30 cases were of leukoplakia and 30 cases were of well-differentiated SCC.

## MATERIALS AND METHODS

This retrospective morphometric study was conducted on tissue sections obtained from the biopsy tissue specimens received and from the cases retrieved from archives of Department of Oral Pathology and Microbiology, V. S. Dental College and Hospital, Bangalore.

The control group comprised normal buccal mucosa (10 cases) from healthy adult individuals, irrespective of age and with no habits. The biopsies were obtained from the patients with their consent, from the Department of Oral Surgery, V. S. Dental College and Hospital.

The study group comprised collection of 30 paraffin blocks of cases of oral leukoplakia of the buccal mucosa and 30 paraffin blocks of cases diagnosed with well-differentiated oral SCC of buccal mucosa.

### Inclusion and exclusion criteria

1. The study group comprised the diagnosed cases, irrespective of age and sex, reported from the Department of Oral Pathology, V. S. Dental College and Hospital.
2. Clinically diagnosed cases of leukoplakia confirmed histologically as epithelial dysplasia [based on WHO grading of epithelial dysplasia (1978)] were only included in the study. Mild, moderate and severe dysplastic cases were all considered since our study included only the basal cell layer of the dysplastic epithelium.
3. Oral SCC cases were selected irrespective of the etiological factors, and only histologically well-differentiated oral SCC cases were included (based on Broder’s grading system).
4. For every case, the most representative areas were selected from the sections which could be subjected to the morphometric analysis in the basal cell layer.

Tissue sections of 5 mm thickness were cut using a soft tissue microtome from formalin-fixed, paraffin-embedded tissue blocks. The sections obtained were stained with Harris’s Hematoxylin and Eosin. The stained sections were observed under a microscope for confirmation of the diagnosis by two separate examiners.

### Morphometric technique

After reviewing, the sections were further subjected to morphometric analysis. For morphometric analysis, images were captured using a three-chip CCD camera attached to a trinocular research microscope with a 100× objective. The final image captured on the monitor had a magnification of 1000×. For each section, the selected field included representative cells with largest cells in the basal layer where distinct cellular and nuclear outlines were seen avoiding areas of basal cell hyperplasia and overlapping cells. Histologically identifiable non-keratinocytes such as melanocytes showing clear cell change and inflammatory cells, as well as cells showing degenerative changes, and those undergoing mitoses were not measured. The images were classified, transferred and stored in the computer.

The actual measurements of the morphometric parameters were done using the image analyzer software Image-Proexpress (Media Cybernetics, Silver Spring, MD, USA) after accurate calibration was done using a stage micrometer. No corrections for section thickness or Holmes effect were applied so that the measurements represented the morphological image as seen.

### Details of Morphometric Parameters Studied [Figures 1 and 2]

**Figure 1:**
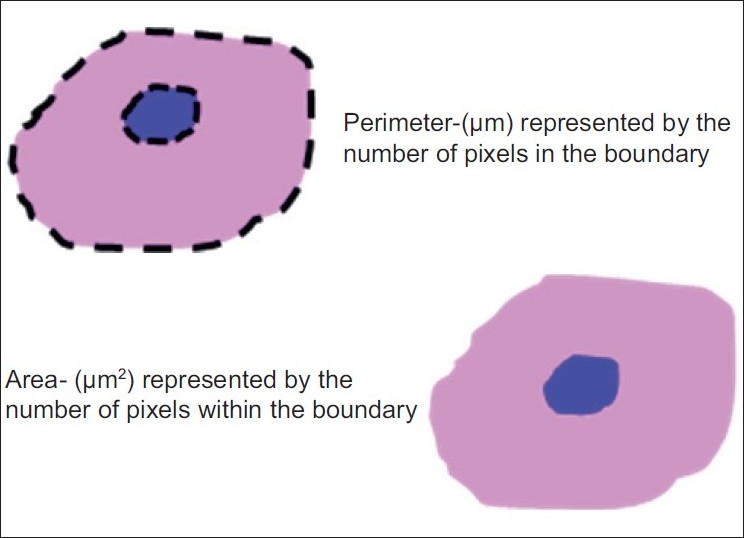
Illustrations and formulas of parameter Size

**Figure 2:**
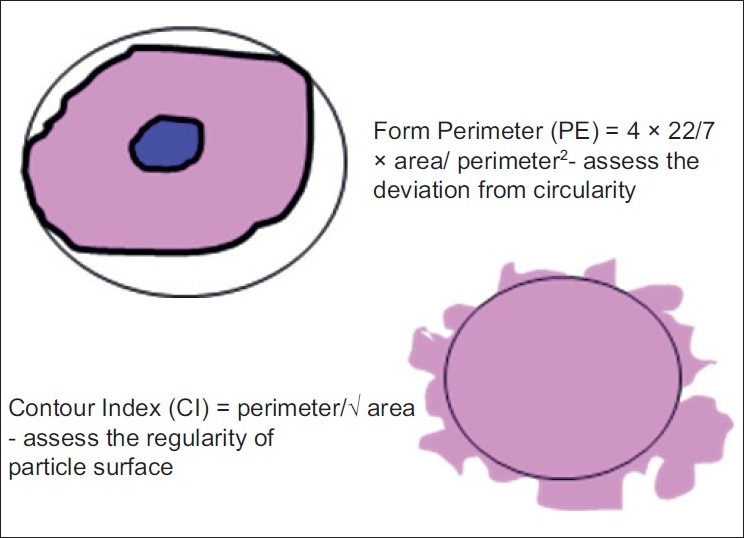
Illustrations and formulas of parameter Shape

#### 1. Size

The size of the cell and its nucleus was measured with the area and perimeter.

### Cell perimeter and nuclear perimeter

It was measured in microns. For measurement, the cell and the nuclear outline were traced and the software automatically calculated the cell and nuclear perimeter (number of boundary pixels detected, converted to micrometer); 5–7 largest cells with clear outline were selected from each field.

### Cell area and nuclear area

It was measured in square microns when the perimeter was traced; the software automatically calculated the CA (number of pixels detected, converted to micrometer^2^).

From the CP, CA, NP and NA, the other morphometric parameters i.e., shape and nuclear–cytoplasmic ratio, were calculated.

#### 2. Shape

The shape was measured using two variables.

- “Form perimeter (PE)” which assesses the deviation from circularity. Using standard formula, a circle has a value of 1 and an ellipse or an irregular structure is less than 1.
  Cellular form PE=4 × 22/7 × C area/C perimeter^2^
  Nuclear form PE=4 × 22/7 × N area/N perimeter^2^
- Contour index (CI) was the other shape measuring variable used to measure the regularity of the particle surface. A value of 3.54 is obtained for a circle by using standard formula. This formula was applied to the cellular and nuclear values.
  Cellular CI=C perimeter/√C area
  Nuclear CI=N perimeter/√N area

Higher values are obtained with the increase in the indentations or convolutions of the outline.

#### 3. N:C ratio

The nuclear–cytoplasmic ratio was the other parameter which was calculated by using the formula

N:C ratio=N area/(C area – N area).

All the measurements were saved in Microsoft excel for further statistical analysis.

## RESULTS

Approximately 7000 measurements were carried out [Table 1] and the results were then represented as mean area and perimeter [Table 2] and the data collected in this study were analyzed statistically by computing descriptive statistics, viz., percentage, mean, standard deviation, standard error of mean, 95% confidence interval for mean values obtained in microns as shown in the tables for various morphometric variables. The differences in the control group and study groups for various diagnostic variables were compared by means of analysis of variance (ANOVA) and Student’s *t*-test for independent samples, wherever applicable, and are shown in Tables 3–7.

**Table 1:** Morphometric measurements carried out

Total sections: 10 normal buccal mucosa + 30 leukoplakia + 30 oral SCC	70 sections
Number of microscopic fields per section	5–10 fields
Cells per field (basal layer)	5–7 cells
Total cells per section	[(5–7 cells) × (5–10 fields)]
Total cells measured	3500 approx.
Total nuclei measured	3500 approx.
Total number of measurements	7000 approx.

**Table 2:** Morphometry summary data

	Leukoplakia				Well-differentiated SCC				Normal buccal mucosa			
	Cell		Nucleus		Cell		Nucleus		Cell		Nucleus	
	Area	Perimeter	Area	Perimeter	Area	Perimeter	Area	Perimeter	Area	Perimeter	Area	Perimeter
1	128.92	51.056	64.651	35.488	155.34	61.765	81.568	53.127	70.549	31.89	25.14	18.444
2	128.43	49.719	62.042	34.333	148.45	56.874	78.433	49.043	73.145	29.133	20.056	15.177
3	122.01	49.092	58.432	31.654	154.23	52.457	68.649	44.678	70.389	28.256	21.558	15.667
4	120.57	47.886	60.324	32.423	137.12	50.396	79.634	39.545	72.266	31.138	32.961	19.706
5	130.42	48.654	62.348	33.865	154.48	63.743	79.363	41.16	82.639	32.584	26.461	18.353
6	118.24	42.123	49.184	29.958	165.24	66.785	82.043	52.787	85.763	31.181	26.177	16.896
7	120.43	42.433	56.645	25.643	178.24	70.613	85.433	46.121	82.629	32.022	30.203	21.116
8	134.26	48.955	72.432	38.423	175.32	58.953	67.789	49.325	83.811	32.268	32.233	20.762
9	119.54	41.564	69.763	35.312	148.23	56.786	72.433	47.228	68.433	24.543	27.798	18.412
10	121.43	51.044	64.959	34.569	143.46	44.867	68.244	34.489	88.976	31.263	27.59	17.836
11	115.43	44.108	52.639	31.432	147.43	55.856	64.246	30.226				
12	114.62	40.948	55.234	27.538	154.05	48.732	81.942	38.772				
13	134.43	54.533	64.675	36.101	138.19	46.257	69.187	33.923				
14	124.43	55.665	60.326	34.426	139.43	42.601	80.516	37.231				
15	112.43	42.243	51.235	32.243	146.43	43.476	68.278	36.452				
16	133.94	51.607	65.787	35.914	173.23	55.787	83.343	47.306				
17	118.46	42.264	55.235	29.343	139.54	54.68	76.439	35.069				
18	134.54	53.569	58.886	37.585	171.34	57.054	72.433	39.211				
19	116.54	45.765	50.234	30.342	157.43	51.299	77.343	39.913				
20	126.64	51.718	51.432	45.581	155.43	50.309	82.456	41.105				
21	121.54	43.947	65.199	35.753	143.46	57.46	68.743	32.456				
22	114.33	45.433	56.178	33.126	166.25	56.598	68.346	35.343				
23	128.43	44.432	56.991	32.764	164.97	51.897	82.264	34.236				
24	114.96	46.277	61.614	32.412	165.69	49.136	72.428	39.253				
25	132.77	49.575	77.286	38.096	163.24	47.981	81.246	40.197				
26	131.82	49.211	60.576	35.078	137.24	46.753	68.234	37.163				
27	118.43	47.887	56.432	32.324	146.86	47.684	68.471	30.324				
28	112.34	54.333	44.232	53.88	143.56	47.006	75.839	37.163				
29	122.45	55.601	54.324	29.543	150.48	52.032	61.433	46.126				
30	130.55	56.796	62.784	32.654	143.9	42.895	67.343	33.896				

**Table 3:** Analysis of variance of nuclear and cytoplasmic areas

Area	Source of variation	Sum of squares	df	Mean square	*F* ratio	*P* value
Nucleus	Between groups	17,064.426	2	8532.213	193.509	<0.0001
	Within groups	2954.169	67	44.092		
	Total	20,018.595	69			
Cell	Between groups	46,560.968	2	23,280.484	240.092	<0.0001

**Table 4:** Statistical inference based on independent Student’s *t*-test

Area	Diagnostic groups	*t* value	df	*P* value
Nucleus	Leukoplakia and normal buccal mucosa	13.509	38	<0.0001
	SCC and normal buccal mucosa	20.875	38	<0.0001
	Leukoplakia and SCC	8.403	58	<0.0001
Cytoplasm	Leukoplakia and normal buccal mucosa	17.015	38	<0.0001
	SCC and normal buccal mucosa	18.367	38	<0.0001
	Leukoplakia and SCC	11.869	58	<0.0001

**Table 5:** Analysis of variance of nuclear and cytoplasmic “form PE”

CPE	Source of variation	Sum of squares	df	Mean square	*F* ratio	*P* value
Nucleus	Between groups	1.290	2	0.645	27.693	<0.0001
	Within groups	1.560	67	0.023		
	Total	2.850	69			
Cell	Between groups	1.189	2	0.594	m30.114	<0.00m01
	Within groups	1.323	67	0.020		
	Total	2.511	69			

**Table 6:** Analysis of variance of nuclear and cytoplasmic contour index (CI)

CI	Source of variation	Sum of squares	df	Mean square	*F* ratio	*P* value
Nucleus	Between groups	9.847	2	4.924	9.681	<0.0001
	Within groups	34.077	67	0.509		
	Total	43.924	69			
Cell	Between groups	6.308	2	3.154	18.122	<0.0001
	Within groups	11.660	67	0.174		
	Total	17.968	69			

**Table 7:** Analysis of variance of nuclear–cytoplasmic ratio

Source of variation	Sum of squares	df	Mean square	*F* ratio	*P* value
Between groups	1.383	2	0.692	21.862	<0.0001
Within groups	2.120	67	0.032		
Total	3.503	69			

Mann–Whitney U-test and Kruskal–Wallis test were used where the data were found to be asymmetrical. The results were considered statistically significant whenever *P*≤0.05.

**Figure 3:**
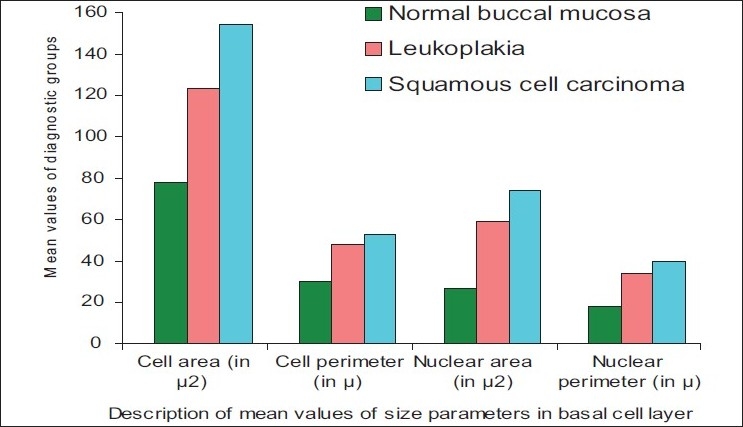
Bar graph showing the description of mean values in the basal cell layer

The results showed that the nuclear area and perimeter increased steadily from normal buccal mucosa to leukoplakia and reached the highest value in well-differentiated SCC. The mean values of the whole cell showed similar trends with increased values of cellular area and perimeter from normal buccal mucosa to leukoplakia to well-differentiated SCC [Figure 3].

**Figure 4:**
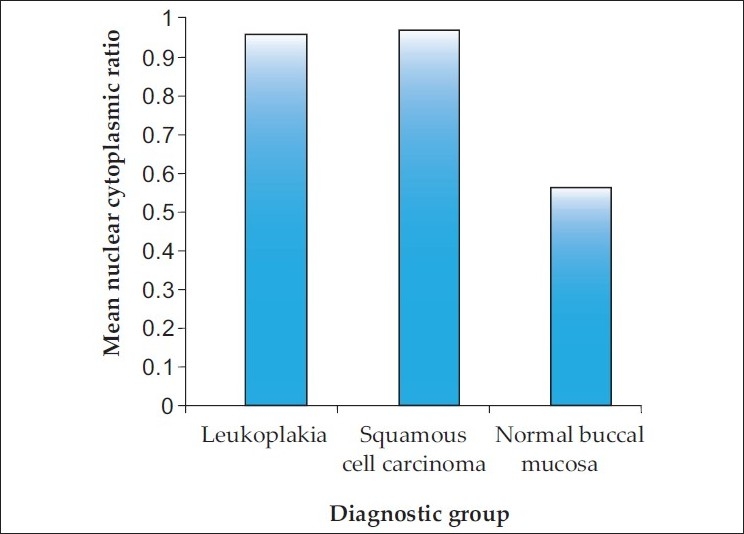
Bar graph showing the mean N:C ratio in the diagnostic groups

There was increase in the mean N:C ratio between normal buccal mucosa and leukoplakia and between normal buccal mucosa and SCC [Figure 4].

The nonparametric Mann–Whitney U-test was used to evaluate the independent pairwise difference between the diagnostic groups of nuclear “form PE”. It was observed that there was a highly statistical significance between normal buccal mucosa and leukoplakia (*Z*=4.46, *P*<0.0001) as well as between normal buccal mucosa and SCC (*Z*=4.56, *P*<0.0001). However, there was no statistically significant result between leukoplakia and SCC (*Z*=1.36, *P*>0.174) for nuclear “form PE”. The results were similar for cellular “form PE”. The Kruskal–Wallis test was used to test the changes in more than two independent groups. Both in nuclear “form PE” and cellular “form PE”, the Kruskal–Wallis test showed that the diagnostic groups were independently distributed (χ^2^=29.934, *P*<0.0001).

The Mann–Whitney test showed statistical significance in nuclear “CI” in the groups between normal buccal mucosa and leukoplakia (*Z*=4.46, *P*<0.0001), and normal buccal mucosa and SCC (*Z*=4.56, *P*<0.0001). There was no statistical significance in nuclear “CI” between leukoplakia and SCC (*Z*=1.36, *P*>0.174). For cellular “CI” also, the results were similar.

However, it could be observed by applying the Kruskal–Wallis test that the N:C ratios between the three diagnostic groups were independently distributed (χ^2^=22.17; df=2, *P*<0.0001)

## DISCUSSION

Oral SCC is the most common malignant neoplasm arising from the mucosal epithelium of the oral cavity. While its incidence is relatively low in the western countries, there are some important exceptions to this trend. In the Indian subcontinent and in other parts of Asia, it remains one of the most common forms of cancer.[15] This is a paradoxical finding since most cases are preceded by readily detectable mucosal change, most often red or white patches.[16] The diagnosis of precancers is primarily based on clinical morphology and its grading on histology (*dysplasia*). The presence of epithelial dysplasia may be even more important in predicting the malignant development than the clinical characteristics.

Three major problems, however, are attached to the importance of epithelial dysplasia in predicting the malignant development:

1. The diagnosis is essentially subjective.
2. Not all lesions exhibiting dysplasia will eventually become malignant.
3. Carcinoma can develop from lesions in which epithelial dysplasia was not diagnosed in previous biopsies.

There is, therefore, a substantial need to improve the histologic assessment of epithelial dysplasia. Of late, interest has turned toward applying sophisticated technique of computer-assisted morphometry to investigate the cellular and nuclear changes in correlation with the histological behavior of the lesions. It is a method of assessing the computerized images of histological stained sections. The results have been more reliable, objective and reproducible.

Many investigators have evaluated the use of nuclear morphometry for grading and for predicting prognosis in esophageal, laryngeal, renal, bladder, breast, prostrate and colonic carcinomas. Attempts have also been made to apply morphometry to normal oral epithelium and also in various oral mucosal diseases like oral submucous fibrosis, leukoplakia, lichen planus, SCC, epithelial dysplasia, and traumatic keratosis.

Boysen and Reith[17] in their study on nasal mucosa in nickel workers have also reported a progressive increase in basal cellular and nuclear areas from pseudostratified columnar epithelium, through metaplastic epithelium, to their highest values in dysplastic epithelium. It could be observed in our study also that there was a steady increase in the basal cellular and nuclear size dimensions from normal buccal mucosa to leukoplakia lesions, indicating more biologic activity in the nucleus and the cytoplasm.

Eveson and MacDonald[18,19] observed that after the application of carcinogens to hamster cheek pouch epithelium, there was progressive increase both in size (represented by mean cell area) and number of progenitor cells. Similar changes have been described in the epithelia after application of turpentine, chemical carcinogens, and also during the menstrual cycle. These findings support our observation of increase in cellular area in the progenitor cells.

Saku and Sato[20] reported an increase in the proportion of the cells in the hyperdiploid to the hypertetraploid range in oral leukoplakia. In our study, the NA was almost twice as large in leukoplakia compared to the normal buccal mucosa.

Shabana *et al*.[14] studied the size and shape of the cells in the basal cell layer of the oral epithelium in 100 specimens from oral mucosa by using an interactive image analysis system (IBAS-1). Four groups of white lesions (traumatic keratosis, lichen planus, leukoplakia, and a “risk group”) in addition to two control groups (normal mucosa and SCC) were studied retrospectively. The results showed a progressive increase in the dimensions (area, perimeter, and maximum diameter) of the nuclei from normal mucosa through traumatic keratosis, lichen planus, leukoplakia and the “risk group” to carcinoma, with considerable differences. The nucleus in SCC was twice as large as in normal mucosa. A substantial increase in the dimensions of both the cell and the nucleus was found in the “risk group”. The nuclear:cytoplasmic ratio, contrary to what might have been anticipated in risk lesions, did not show considerable differences between the diagnostic groups. Furthermore, it was slightly decreased in the “risk group” compared with the normal mucosa. The shape factors (form PE and CI) seemed to be less helpful in the identification of the “risk group”. They concluded that the size of the basal cell and its nucleus can be of diagnostic value for lesions with a high risk of malignant transformation.

Our results were significant for the morphometric parameter, size. The values of NP and NA, CP and CA increased gradually from the normal buccal mucosa to leukoplakia, reaching the highest value in SCC. There was statistically significant difference in the nuclear and cellular areas to differentiate between leukoplakia and SCC. The morphometric parameter, size, was hence useful to differentiate between normal, potentially malignant leukoplakia and the malignant SCC. Two variables which were used to study the shape, “form PE” and “CI”, showed significant difference between normal buccal mucosa and leukoplakia and between normal buccal mucosa and SCC. However, this parameter was of no use to differentiate between leukoplakia and SCC. In our study, the mean±SD (mean) of N:C ratio was 0.941±0.182, whereas in the study by Shabana *et al*., the mean±SD (mean) was 0.809±0.215. The N:C ratio was slightly higher in our study denoting more NA compared to the cytoplasmic area. It can be observed that though there was a significant difference statistically in the N:C ratio between normal buccal mucosa and SCC, it was not seen between leukoplakia and SCC.

Cowpe *et al*.[21] found that tissues undergoing malignant transformation typically showed a reduction in CA before the reduction in NA, using semiautomatic image analysis techniques. They also suggested that samples of healthy mucosa from the same patient provide the best control. In an earlier study, they demonstrated that exfoliative cytology is capable of detecting malignant changes, through estimation of NA/CA using the planimeter method in Papanicolaou-stained smears.[22]

Truelson *et al*.[23] noted that nuclear DNA content and nuclear area were better indicators of the biologic aggressiveness of cancer in laryngeal cancers. In the study by Jin *et al*.,[24] the mean±SD of N:C ratio of islands of invasive SCC was 0.650±0.207. Even though their value increased from that of dysplastic epithelium (0.558±0.101), there was no significant difference statistically between the two groups. They concluded that this parameter was not useful to distinguish between premalignant and malignant lesions. Our results showed an increase in N:C ratio of the oral basal cells in leukoplakia and SCC compared to normal buccal mucosa, but the difference was not significant between leukoplakia and SCC.

Ramaesh *et al*.[25,26] used cytomorphometric techniques to assess nuclear diameter (ND) and cytoplasmic diameter (CD) in normal oral mucosa, in dysplastic lesions and in SCCs. They found that CD was highest in normal mucosa, lower in dysplastic lesions, and lowest in SCCs. By contrast, ND was the lowest in normal mucosa, higher in dysplastic lesions, and highest in SCCs. These studies suggested that reduced nuclear size and increased cytoplasm size are useful early indicators of malignant transformation, and thus, exfoliative cytology is of value for monitoring clinically suspected lesions and for early detection of malignancy.

Mollaoglu *et al*.[27] used an advanced imaging system, Seescan TV image analysis system (TVIAS), and could successfully identify dysplastic and malignant cells in oral smears containing large amounts of normal cells, on the basis of both integrated optical density (IOD) and NA values. There was a significant increase (*P*<0.001) in mean IOD for nuclei in smears from dysplastic lesions and carcinomas as compared with normal smears of 50 Feulgen-stained nuclei which were measured in smears collected from normal oral mucosa (*n*=6), lesions displaying epithelial dysplasia (*n*=5) and invasive SCC (*n*=5).

Zo Pektas *et al*,[28] used cytomorphometric measurements and nuclear Feulgen DNA content (DNA ploidy) analysis via oral brush biopsy in oral mucosal smears (*n*=44) from patients (*n*=22) presenting with various oral lesions using a cytobrush immediately before biopsy. They concluded that cytomorphometric analysis via oral brush biopsy, a valuable adjunct to biopsy for identification of premalignant and early stage cancerous oral lesions, is a rapid and minimally invasive procedure with high specificity and sensitivity rates, requiring no topical or local anesthetic.

Natarajan *et al*.[29] attempted an objective and reproducible evaluation of mitotic activity and nuclear morphometric factors in their study of 30 patients of oral SCC with a view to predicting local relapse and survival. Various nuclear parameters and volume-corrected mitotic index were calculated and compared with the recurrence and death of the study group (*n*=30), and the effectiveness of the M/V index in predicting the biological behavior and the outcome of oral SCC patients was determined.

### Observations drawn from our study and few suggestions

1. More quantitative morphometric parameters like volume densities, texture analysis, state of nucleus described subjectively as coarse, spotty, dispersed or clumped have to be analyzed to determine the relationship of these atypical features to subsequent malignant transformation.
2. Other quantifiable features of epithelial dysplasia, already defined, have to be assessed to know whether they have greater or lesser diagnostic significance using image analysis.
3. Epidemiological studies have shown that candidal leukoplakia and proliferative verrucous leukoplakia have greater malignant potential. Further studies regarding these pathological groups are necessary to substantiate this claim.
4. Rigorous assessments have to be performed using multivariate statistics to check the validity of the results obtained from large quantitative data.
  - To summarize, the objectives of this morphometric analyses were to study the a) size, b) shape, and c) N:C ratio of the basal cells and nuclei in normal mucosa, leukoplakia and well-differentiated SCC using image analysis. The results have revealed the following:The morphometric parameter **size** was useful to differentiate basal cells in normal mucosa, leukoplakia and SCC.
  - The morphometric parameter shape was useful to differentiate between normal mucosa and leukoplakia but not between leukoplakia and SCC.
  - The N:C ratio parameter was useful to differentiate between normal mucosa and leukoplakia but not between leukoplakia and SCC.

In conclusion, the increase in basal cellular and nuclear size in leukoplakia has shown it to be more prone for malignant change, and therefore, their measurements may provide an objective means for the assessment of epithelial dysplasia and to predict their malignant potential. Techniques of image analysis offer an opportunity to quantify the nuclear and cell changes associated with malignancy and provide an objective basis for grading dysplasia and tumors. Overall, these results have shown that the quantitative histomorphometric techniques can detect features that may be overlooked by routine histological examination.
